# Risk factors for postoperative delirium in patients undergoing microvascular decompression

**DOI:** 10.1371/journal.pone.0215374

**Published:** 2019-04-18

**Authors:** Zhenhua He, Huijuan Cheng, Haiyang Wu, Guodong Sun, Jingmin Yuan

**Affiliations:** 1 Department of Neurosurgery, Lanzhou University Second Hospital, Lanzhou, Gansu Province, People’s Republic of China; 2 Gansu Provincial Key Laboratory of Digestive System Tumors, Lanzhou University Second Hospital, Lanzhou, Gansu Province, People’s Republic of China; 3 Department of Pain, Lanzhou University Second Hospital, Lanzhou, Gansu Province, People’s Republic of China; University Magna Graecia of Catanzaro, ITALY

## Abstract

This study is to identify the risk factors for postoperative delirium (PODE) in patients undergoing microvascular decompression (MVD) for the treatment of primary cranial nerve disorders. We retrospectively reviewed the data of 912 patients (354 men, 558 women) with primary cranial nerve disorders (trigeminal neuralgia, 602 patients; hemifacial spasm, 296 patients; glossopharyngeal neuralgia, 14 patients) who underwent MVD in the Neurosurgery Department of Lanzhou University Second Hospital between July 2007 and June 2018. Potential risk factors for PODE were identified using univariate and multivariate stepwise logistic regression analysis.Of the 912 patients, 221 (24.2%) patients developed PODE. Patients with PODE were significantly older and significantly more likely to be male than patients without PODE. A history of hypertension, preoperative carbamazepine therapy, and postoperative sleep disturbance and tension pneumocephalus were independently associated with PODE. Variables such as body-mass index, smoking and drinking habits, cardiac disease, diabetes mellitus, cerebrovascular disease, mean operative time, affected vessel, mean blood loss, postoperative intensive care unit stay, postoperative fever (>38°C), and routine laboratory results were not associated with PODE in our patients.PODE is a common complication after MVD, and is associated with multiple risk factors, including old age, male sex, hypertension, preoperative carbamazepine use, postoperative sleep disturbance, and tension pneumocephalus.

## Introduction

Microvascular decompression (MVD) is widely recognized as the neurosurgical treatment of choice for patients with primary cranial nerve disorders, including trigeminal neuralgia, hemifacial spasm, and glossopharyngeal neuralgia, as well as various neurovascular compressions[1]. However, delirium is a common complication after MVD surgery, with reported incidence rates of 14.9%–27.3% [2]. This postoperative delirium (PODE) is an acute but transient organic brain syndrome characterized by inattention and altered levels of consciousness [3]. The development of PODE after MVD procedures tends to prolong hospital stays and increase medical costs as well as morbidity and mortality [4]. However, few studies have focused on PODE in patients undergoing MVD surgery [2, 5], and the exact pathogenesis of this complication remains poorly understood. A deeper understanding of the risk factors for PODE is required to identify at-risk patients as well as possible therapeutic interventions. Therefore, in this study, we aimed to identify the independent risk factors for PODE after MVD surgery in a large cohort of patients with primary cranial nerve disorders. We hope that our findings will indicate possible clinical interventions that might minimize the risk of PODE in this patient population.

## Methods

### Patients

This retrospective study involved patients with primary cranial nerve disorders who underwent MVD procedures in the Neurosurgery Department of Lanzhou University Second Hospital between July 2007 and June 2018. None of the patients had any abnormalities on preoperative imaging, such as multiple sclerosis, vascular malformation, and tumor. Furthermore, no patient had a history of psychiatric disease. All patients underwent MVD under general anesthesia by the same senior neurosurgeon performing the same surgical technique.

This study was approved by the ethics committee of Lanzhou University Second Hospital Medical (2018A-005) and was conducted in accordance with the principles of the Declaration of Helsinki. The requirement for individual informed consent was waived by the ethics committee, as this was a retrospective study of electronic medical records.

### Diagnostic criteria for PODE

For all patients, delirium observation screening scores were calculated three times a day, from day 2 to day 5 after the surgery [6]. For patients with scores of three or greater, a psychiatrist consult was requested to confirm the diagnosis of PODE according to the Diagnostic and Statistical Manual of Mental Disorders, 5^th^ edition (DSM V, 2013) [7]. Patients who did not develop delirium during the first 5 days after the surgery were included in the non-PODE group.

### Data collection

We collected data on pre-, intra-, and postoperative variables. The preoperative variables included age, sex, body-mass index, smoking (tobacco use within 3 months before surgery), drinking (alcohol use within 3 months before surgery), cardiac disease (coronary artery disease, myocardial infarction, and unstable angina), hypertension (blood pressure > 140/90 mm Hg), diabetes mellitus, and cerebrovascular disease. The intraoperative variables included mean operative time, affected vessels, and mean blood loss. The postoperative variables included intensive care unit (ICU) stay, fever (body temperature > 38°C), and sleep disturbance. Sleep quality was assessed using the Pittsburgh Sleep Quality Index (PSQI). The PSQI domain scores were summed to obtain a global count score, which varied from 0 to 21, with higher scores indicating worse disturbance. We defined sleep disturbance as a PSQI score > 5 [8].

We also recorded postoperative laboratory data measured within 3 days of the surgery: hemoglobin, white blood cell count, serum sodium, serum potassium, blood urea nitrogen (BUN), and creatinine. Additionally, we noted the following details of carbamazepine (CBZ) treatment: history of preoperative CBZ therapy, preoperative CBZ dose, mean duration of CBZ use, and serum CBZ levels at 24 h before and after the surgery. We also noted the findings of computed tomography (CT) of the brain performed within 24 h after the surgery.

### Statistical analysis

All statistical analyses were conducted using the Statistical Package for the Social Sciences (IBM SPSS version 24.0). The chi-square test and Fisher exact test were used for count data, while variance analysis and the *t*-test were used for measurement data. Univariate stepwise logistic regression analysis was performed using variables with P values of <0.1. Variables that were statistically significant in the univariate analysis (P < 0.05) were subjected to multivariate regression analysis. Odds ratios (ORs) were calculated and were considered to be statistically insignificant if the 95% confidence interval (CI) was 1.

## Results

### General information

A total of 912 patients, consisting of 558 women and 354 men, were included in this study. The primary cranial nerve disorders diagnosed in these patients were as follows: trigeminal neuralgia, 602 patients; hemifacial spasm, 296 patients; and glossopharyngeal neuralgia, 14 patients. Preoperative CBZ treatment was administered to 668 patients. PODE occurred in 221 of the 912 patients (24.2%). Delirium was observed a mean of 3.1 days after the surgery, and the mean duration of delirium was 2.4 days (range, 1–4 days).

### Preoperative factors

Patients who developed PODE were significantly older than patients who did not (61.01 ± 11.70 vs. 59.17 ± 10.21 years, P = 0.036). The incidence of PODE was significantly higher in male patients than in female patients (35% vs. 17.3%, OR: 2.66, 95% CI: 1.91–3.71). Additionally, patients with PODE had higher rates of hypertension than did patients without PODE (29.9% vs. 15.9%, OR: 2.25, 95% CI: 1.53–3.30). BMI, smoking and drinking habits, and the rates of cardiac disease, diabetes mellitus, and cerebrovascular disease did not significantly differ between the PODE and non-PODE groups (Table 1). Of the 221 patients who developed PODE, 182 patients (82.4%) had received preoperative CBZ therapy, indicating that CBZ use was significantly associated with the occurrence of PODE (P = 0.006). Furthermore, compared with the non-PODE group, the PODE-group patients had significantly higher preoperative CBZ dosage (708.14 ± 68.12 vs. 599.49 ± 67.38 mg/day, P < 0.001), significantly higher serum CBZ concentration at 24 h before the surgery (6.11 ± 1.21 vs. 5.26 ± 1.05 μg/mL, P < 0.001), and significantly longer duration of CBZ use (5.96 ± 0.77 vs. 4.94 ± 0.63 years, P < 0.001; Table 2).

**Table 1:** Association between preoperative factors and postoperative delirium (PODE).

Variable	PODE (n = 221)	No PODE (n = 691)	P value
Age (years)*	61.01 ± 11.70	59.17 ± 10.21	0.036
BMI (kg/m^2^)*	25.31 ± 2.67	25.19 ± 2.62	0.552
Male sex (n, %)	124 (56.1%)	230 (33.3%)	<0.001
Smoking (n, %)	42 (19.0%)	135 (19.5%)	0.862
Alcohol use (n, %)	28 (12.7%)	100 (14.5%)	0.502
Cardiac disease (n %)	40 (18.1%)	63 (9.1%)	<0.001
Hypertension (n, %)	66 (29.9%)	110 (15.9%)	<0.001
Diabetes mellitus (n, %)	57 (25.8%)	128 (18.5%)	0.019
Cerebrovascular disease (n, %)	79 (35.7%)	181 (26.2%)	0.006

*Values are expressed as mean ± standard deviation

BMI, body-mass index

**Table 2:** Association between carbamazepine (CBZ) treatment and postoperative delirium (PODE).

Variable	PODE (n = 221)	No PODE (n = 691)	P value
Preoperative CBZ therapy (n, %)	182 (82.4%)	506 (73.2%)	0.006
Preoperative CBZ dose (mg)	708.14 ± 68.10	599.49 ± 67.38	<0.001
Duration of CBZ use (years)	5.96 ± 0.77	4.94 ± 0.63	<0.001
Preoperative CBZ (μg/mL)*	6.11 ± 1.21	5.26 ± 1.05	<0.001
Postoperative CBZ (μg/mL)^&^	1.40 ± 0.18	1.42 ± 0.23	0.127

*Serum CBZ level measured 24 h before surgery

^&^Serum CBZ level measured 24 h after surgery

Values are expressed as mean ± standard deviation unless otherwise specified.

### Intraoperative factors

The mean operative time did not differ between the PODE group (156.11 ± 13.86 min) and the non-PODE group (155.13 ± 12.95 min, P = 0.337). During the surgery, neurovascular compression was confirmed in 892 of the 912 patients. The most common vessels affected in the PODE group were as follows: superior cerebellar artery (SCA, 338/602), anterior inferior cerebellar artery (AICA, 116/602), petrosal vein (PV, 61/602), and SCA-PV (31/602) in patients with trigeminal neuralgia; AICA (158/296), PICA (86/296), and vertebral artery (VA, 44/296) in patients with hemifacial spasm; and PICA (9/14) in patients with glossopharyngeal neuralgia. No significant differences in the types of vessels affected were observed between the PODE and non-PODE groups (P > 0.05). The mean blood loss also did not differ between the PODE (328.67 ± 32.08 mL) and non-PODE groups (331.44 ± 30.69 mL, P > 0.05; Table 3).

**Table 3:** Association between intraoperative factors and postoperative delirium (PODE).

Variable	PODE (n = 221)	No PODE (n = 691)	P value
Affected blood vessel			
Trigeminal neuralgia (n = 602)			
SCA	94	242	0.872
AICA	32	85	0.812
Petrosal vein	15	46	0.504
SCA-petrosal vein	7	24	0.472
SCA-AICA	4	11	1.000*
Basilar artery	5	6	0.307*
Arteriole	4	6	0.480*
No vascular compression	9	12	0.130
Hemifacial spasm (n = 296)			
AICA	21	137	0.077
PICA	19	67	0.126
Vertebral artery	8	36	0.805
AICA-vertebral artery	2	6	0.626*
Glossopharyngeal neuralgia (n = 14)			
PICA	1	8	1.000*
AICA	0	3	1.000*
Vertebral artery	0	1	1.000*
PICA-vertebral artery	0	1	1.000*
Mean blood loss (mL)	328.67 ± 32.08	331.44 ± 30.69	0.250
Mean operative time (min)	156.11 ± 13.86	155.13 ± 12.95	0.337

*Fisher exact test

SCA, superior cerebellar artery; AICA, anterior inferior cerebellar artery; PICA, posterior inferior cerebellar artery

### Postoperative factors

**Fig 1:**
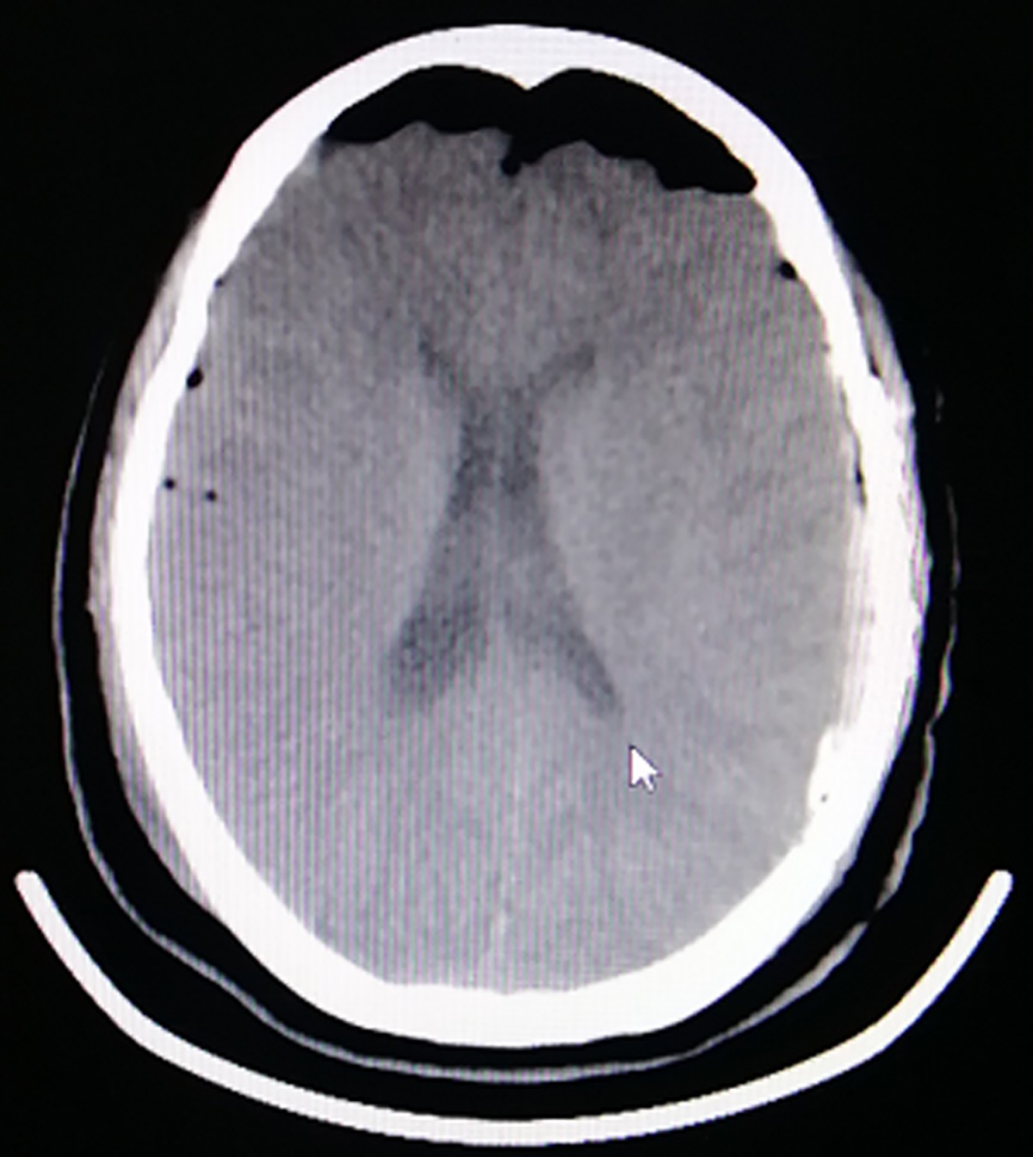
A postoperative computed tomography scan (axial view) showing intracranial air compressing the frontal lobes with widening of the interhemispheric space between the frontal poles, i.e., the Mt. Fuji sign.

The postoperative length of ICU stay was similar in the PODE (2.95 ± 1.10 days) and non-PODE groups (3.04 ± 1.01 days). The incidence of postoperative fever also did not differ between the PODE group (27/221) and the non-PODE group (84/691; Table 4). Sleep disturbance was noted in 20.8% (46/221) of patients in the PODE group and 4.8% (33/691) of patients in the non-PODE group (OR: 4.95, 95% CI: 2.95–8.29). The serum CBZ level at 24 h after the operation did not differ between the two groups (P = 0.127). No significant differences were found in any of the postoperative laboratory data between the two patient groups, including the hemoglobin, white blood cell count, serum Na level, serum K level, BUN, and serum creatinine value (Table 4). Tension pneumocephalus was diagnosed (>65 mL air volume and Mount Fuji sign on CT; Fig 1) in 57 of the 221 patients in the PODE group and 63 of the 691 patients in the non-PODE group (OR: 3.24, 95% CI: 2.10–4.99; Table 5).

**Table 4:** Association between postoperative factors and postoperative delirium (PODE).

Variable	PODE (n = 221)	No PODE (n = 691)	P value
ICU stay (days)	2.95 ± 1.10	3.04 ± 1.01	0.280
Temperature > 38°C (n, %)	27 (12.2%)	84 (12.2%)	0.981
Sleep disturbance (n, %)	46 (20.8%)	33 (4.8%)	<0.001
Mount Fuji sign (n, %)	57 (25.8%)	63 (9.1%)	<0.001
HGB (g/L)	100.73 ± 4.83	100.13 ± 4.37	0.097
WBC (×10^9^/L)	10.88 ± 2.04	10.69 ± 2.23	0.250
Serum Na (mmol/L)	136.95 ± 2.25	136.96 ± 2.44	0.950
Serum K (mmol/L)	3.80 ± 0.31	3.76 ± 0.32	0.069
BUN (mmol/L)	3.99 ± 0.31	4.01 ± 0.41	0.376
CREA (μmol/L)	81.49 ± 2.63	80.26 ± 28.53	0.520

Values are expressed as mean ± standard deviation unless otherwise specified. ICU, intensive care unit; HGB, hemoglobin; WBC, white blood cell count; BUN, blood urea nitrogen; CREA, creatinine

**Table 5:** Multivariate stepwise logistical regression analysis of predictors of postoperative delirium (PODE) in patients undergoing microvascular decompression.

Variable	Regression coefficient	OR (95% CI)	P value
Male sex*	0.980	2.66 (1.91–3.71)	<0.001
Hypertension*	0.810	2.25 (1.53–3.30)	<0.001
Sleep disturbance**	1.599	4.95 (2.95–8.29)	<0.001
Mount Fuji sign**	1.175	3.24 (2.10–4.99)	<0.001

OR, odds ratio; CI, confidence interval

*Preoperative variable

**Postoperative variable

Multivariate regression analysis showed that male sex, a history of hypertension, postoperative sleep disturbance, and tension pneumocephalus were independently associated with PODE (Table 5).

## Discussion

The present study found that PODE is a common complication after MVD surgery for patients with primary cranial nerve disorders. Furthermore, the study identified multiple risk factors for this complication, including older age, male sex, a history of hypertension, postoperative sleep disturbance, postoperative tension pneumocephalus, and preoperative CBZ therapy. The incidence of PODE in our study was 24.2%, which is consistent with a previous report [9].

Several studies have indicated that age and sex are risk factors for PODE [10, 11]. Consistent with this, the present study found that patients with PODE were significantly older and significantly more likely to be male than patients without PODE. A reduction in sensory perception is known to precipitate the cognitive confusion associated with advanced age [12]. Additionally, the body’s primary inflammatory response can lead to neuroinflammation and increase the risk of PODE [13]. Both cognitive function and immunity are poorer among elderly male patients, who are more likely to experience negative emotions due to the surgical wound, unfamiliar environment, and medical instrumentation associated with MVD [14, 15]. Furthermore, encephalatrophy is closely associated with advanced age.

A history of hypertension was an independent predictor of PODE in our study. This finding is consistent with recent pooled evidence on the risk factors for PODE [16]. Patients with cranial nerve disorders who have hypertension usually receive treatment with multiple drugs, such as antihypertensive and antipsychotic drugs, and this has been associated with delirium in the clinical setting [17]. In addition, among hypertensive patients, blood pressure may rise above the optimal mean arterial pressure during the perioperative period, and this has been associated with both the incidence and severity of PODE [18].

Postoperative sleep disturbance, as indicated by the PSQI scores, was significantly more common in the PODE group than in the non-PODE group (20.8% vs. 4.8%, OR: 4.95, 95% CI: 2.95–8.29). Sleep disturbance can cause severe confusion. Patients who experience sleep disturbance may develop negative emotions such as anxiety, loneliness, and irritation, leading to PODE [19]. As mentioned earlier, primary cranial nerve disorders are more common in elderly patients, and the older the age, the worse is the sleep disturbance [20].

Tension pneumocephalus is known to occur after MVD surgery in the posterior fossa [21]. Tension pneumocephalus develops when air enters the intracranial cavity through a one-way valve. In our study, tension pneumocephalus was more common in the PODE group than in the non-PODE group (25.8% vs. 9.1%, OR: 3.24, 95% CI: 2.10–4.99). We defined tension pneumocephalus as an intracranial air volume > 65 mL. The entry of air in the subdural space increases the intracranial pressure and leads to neurological deterioration [22]. The Mount Fuji sign is created by the widening of the interhemispheric space between the tips of the frontal lobes and the partial collapse of the frontal lobes due to the entry of air in the subdural space [23]. These changes impair the functional connectivity of the frontal lobes, which is believed to precede the emergence of delirium [24]. Hence, surgeons must closely monitor older patients with sleep disturbance and the Mount Fuji sign after MVD surgery.

At present, CBZ is the first line of treatment for patients with primary cranial nerve disorders [25, 26]. CBZ is an inhibitor of neuronal membrane sodium channels, and reduces the capacity for high-frequency neuronal firing [27]. In our study, both the dose and duration of preoperative CBZ treatment were significantly associated with PODE. Moreover, the serum CBZ concentration at 24 h before surgery was significantly higher in the PODE group than in the non-PODE group (6.11 ± 1.21 vs. 5.26 ± 1.05 μg/mL, P < 0.001), but the serum CBZ level at 24 h after surgery was nearly identical in the two groups (1.40 ± 0.18 vs. 1.42 ± 0.23 μg/mL, P > 0.05). Therefore, we hypothesized that the rapid elimination of CBZ after surgery was linked with the higher incidence of PODE. Thus, the preoperative dosage and duration of CBZ therapy and the rate of change in the serum CBZ concentration after surgery may be important determinants of PODE. In our experience, to avoid the development of PODE, CBZ should be gradually discontinued before MVD, and 100 mg CBZ should be administered orally after MVD if PODE occurs.

This study has a number of limitations. First, the present study was a single-center observational study. Therefore, some information that might affect the incidence of PODE was unavailable or incomplete, such as cognitive function, use of antipsychotic drugs, and functional status before admission. Second, although we used the DSM V for the diagnosis of delirium, the most appropriate method of screening for delirium in patients undergoing MVD procedures remains unclear.

## Conclusion

PODE is a common complication of MVD surgery among patients with primary cranial nerve disorders, occurring in nearly a quarter of our patients (24.2%). Old age, male sex, a history of hypertension, preoperative CBZ therapy (especially long-term high-dose therapy), postoperative sleep disturbance, and the Mount Fuji sign on postoperative brain CT scans were associated with developing PODE after MVD procedures. Patients with these risk factors should be closely monitored. In addition, the dosage of CBZ should be gradually lowered prior to surgery.

## Supporting information

S1 Table delirium_raw data. The electronic medical records of patients undergoing microvascular decompression.
(XLSX)
